# Exploring virus relationships based on virus-host protein-protein interaction network

**DOI:** 10.1186/1752-0509-5-S3-S11

**Published:** 2011-12-23

**Authors:** Feng Xu, Chen Zhao, Yuhua Li, Jiang Li, Youping Deng, Tieliu Shi

**Affiliations:** 1 The Center for Bioinformatics and Computational Biology and the Institute of Biomedical Sciences, the School of Life Science, East China Normal University, Shanghai, 200241, China; 2 College of Life Sciences, the Northeast Forestry University, Harbin, Heilongjiang, 150040, China; 3 Department of Internal Medicine, Rush University Medical Center, Chicago, Illinois 60612, USA

## Abstract

**Background:**

Currently, several systems have been proposed to classify viruses and indicate the relationships between different ones, though each system has its limitations because of the complexity of viral origins and their rapid evolution rate. We hereby propose a new method to explore the relationships between different viruses.

**Method:**

A new method, which is based on the virus-host protein-protein interaction network, is proposed in this paper to categorize viruses. The distances between 114 human viruses, including 48 HIV-1 and HIV-2 viruses, are estimated according to the protein-protein interaction network between these viruses and humans.

**Conclusions/significance:**

The results demonstrated that our method can disclose not only relationships consistent with the taxonomic results of currently used systems of classification but also the potential relationships that the current virus classification systems have not revealed. Moreover, the method points to a new direction where the functional relationships between viruses and hosts can be used to explore the virus relationships on a systematic level.

## Introduction

Viruses can be classified according to different aspects [1,2], such as their geometry, whether they have an envelope, the identity of the host organism they can infect, the mode of transmission, or the type of disease they cause. One of the widely accepted and useful classification systems is based on the combination of their nucleic acid (DNA or RNA), strandedness (single-stranded or double-stranded), sense, and method of replication. This classification system was proposed by David Baltimore [3]. However, the system concerning the diseases caused by the viruses or the morphology of the viruses is not generally accepted, because different viruses could cause the same disease and their morphologies could look very similar under the microscope. It is the instability of the classification system based on viral characteristics that keep these kinds of taxonomy systems from general acceptance. The Baltimore classification offers a way to classify viruses in a given category and behaves in a definite pattern. Meanwhile, the International Committee on Taxonomy of viruses (ICTV) [4-6] has also devised and implemented rules for the classification of viruses. The ICTV system shares many features with the taxonomy system of cellular organisms, such as structure, etc. This classification uses the regular succession of Order, Family, Subfamily, Genus, and Species. Particularly, the code of nomenclature regulated by the International Committee on Taxonomy of Viruses differs from the others on several points. Most notably, names of orders and families are italicized, and species names are not binomial, but instead, they generally take the form of [Host] virus. Up to now, 84 families and more than 2,000 species of virus have been defined by the ICTV classification system. In addition, other virus classification systems, such as Holmes classification [7], LHT System of Virus Classification [8], and Casjens and Kings classification [9] of viruses, have been also proposed.

All of the three main classification systems mentioned above, the Baltimore classification, LHT System, and Casjens and Kings Classification, are based on certain chemical or physical characteristics of viruses. On the other hand, the ICTV classification system is based on the hypothesis that the members of the order might have a common ancestor. However, sixty-four of the total 84 families in the ICTV classification system are still unplaced. The Holmes classification sorts viruses into *Phaginae *(attacks bacteria), *Phytophaginae *(attacks plants), and *Zoophaginae *(attacks animals) according to the host type. These classifications are accepted by some virologists, but not all the virologists are satisfied with the current virus classification systems [10]. Some of them even do not take the information of virus taxonomy into consideration while doing their research because of the limitations of each classification method mentioned above. As the current taxonomies of the viruses do not reflect the phylogeny relationship of different viruses [11], it is hard to build a classification system to satisfy all virologists. However, different methods could be set up to show the relationship between different viruses, which might be helpful to virologists.

A virus needs to use the DNA replication and protein synthetic systems of its host to complete its life cycle and proliferation. Therefore, some viruses exhibit strong co-evolutionary relationships with their mammalian host organisms [12,13]. The study of co-evolution dates back to over 40 years ago [14]. The co-evolution models, including "gene-for-gene", "matching allele", and "matching genotype", have already been proposed [15-18]. In addition, evidence of co-evolution is available in both the temporal and the spatial patterns [14]. As essential viral proteins usually interact with their host proteins, we extend the hypothesis "function association - guilty by association" in an organism to the protein interactions between the organisms and explore the relationships between different viruses by examining the virus-host protein-protein interaction network. The Gene Ontology, which is comprised of terms in a hierarchical tree structure and is adopted for the gene function assignment of most studied organisms, is now extending its realm to the field of microbial annotation. The project of PAMGO (Plant-Associated Microbe Gene Ontology) has already extended the GO system to describe various processes related to microbe-host interactions. Currently, the project has assigned more than 800 new GO terms for microbe-host interaction or other symbiotic interactions [19]. The controlled vocabulary of GO offers scientific researchers a consistent framework to gain and process biological information, thus minimizing the trouble coupled with the variations in human language and its inconsistency across different research communities. In this paper, we hypothesize that the relationship between viruses can be explored through the functional relationship of their proteins' interacting partners from their hosts. To demonstrate this, we used the viruses and human as the model since the protein-protein interaction (PPI) data between viruses and human have been accumulated significantly more than such data between viruses and other organisms. First, the PPI data between viruses and human are collected. Next, the relationship between distinct viruses is inferred based on the functional similarity between different GO terms of their proteins' interacting partners in human. Our estimation of the relationships between different viruses shows a different perspective to the relations of viruses that attack the same host and could be regarded as complementary to the traditional virus classification systems.

## Results and discussion

**Figure 1:**
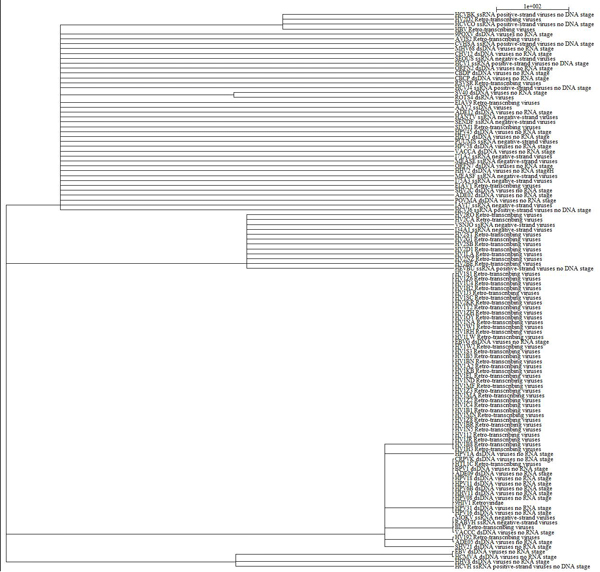
**Virus relationship tree based on molecular function**. 114 viruses are shown here. The name of each item is composed of two parts. The first part is the Uniprot ID of a virus, and the second part of the item name is the basic information of the virus. The length of each branch represents the distance between distinct viruses.

Our method based on the virus-host protein-protein interaction network has shown us the relationships among different viruses according to molecular function. As it was demonstrated in the molecular function tree (Figure 1), 114 viruses whose proteins interacted with human proteins were displayed. In the Figure 1, HIV-1 viruses that belong to the retro-transcribing virus category were generally grouped together. Furthermore, some dsDNA viruses, including CRPVK, BPV1, HPV18, HPV11, HPV6B, HHV11, HPV11, ADE09, and HPV16, were also grouped together. All of these placements are consistent with previous virus taxonomy, which illustrates that the method based on the virus-host protein-protein interaction network has the ability to disclose the similarities among the same type of viruses. However, in the very same figure, it reveals that some viruses possessed different nucleic acid types and were clustered together, an outcome that conflicted with the Baltimore classification method. This phenomenon signifies at least two points. First, as our method of cataloging viruses relies on the protein-protein interaction data between each virus and its host, more available PPI data leads to better elucidation of the relationship. Since the protein-protein interaction data between most viruses and human are incomplete, and some viruses have more PPI data available than others, the unbalanced protein-protein interaction data could potentially have an effect on the tree structure. This conclusion is supported by the finding that the relationships between viruses with more PPI data have much better consistency with the results of other classification systems. Second, by exploring the virus-human protein-protein interaction network, we found that viruses of different types can target the same human proteins. For example, the proteins (PHOSP_RABVH and Q910M0_MOKV) of RABVH and MOKV, which belong to single-stranded RNA viruses, interact with the human protein that has the GO annotation Dynein light chain 1 (DYL1_HUMAN), cytoplasmic, while the protein (VA36_VACCC) of VACCC, which is a double-stranded DNA virus, also interacts with the similar human protein (KLC2_HUMAN, Kinesin light chain). All of the interactions in our network are from Dyer et al. (23) and Pathogen Interaction Gateway, which were experimentally supported. The functional similarity of the target human proteins might narrow the distance of different types of virus. These results may indicate the relationship between viruses of different types. Due to the complexity of the virus classification system and the rapid evolution rate of viruses, there could be some hidden relationship between those dissimilar viruses. For example, different viruses may evolve to target the same cellular processes, called convergent evolution. Traditional taxonomy systems, which merely take chemical or physical characteristics into consideration, might not have the ability to reveal this kind of hidden relationship. Moreover, we would like to point out that our method does not reflect the phylogenic relationships between different viruses. Instead, our classification could disclose the similarity of potential pathogenic mechanisms of distinct viruses, which could be helpful to the treatment of related disease. Our new method could be regarded as complementary for the virologist to discover the relationship between viruses of different types, especially when the potential mechanism that our method may disclose is taken into consideration.

**Figure 2:**
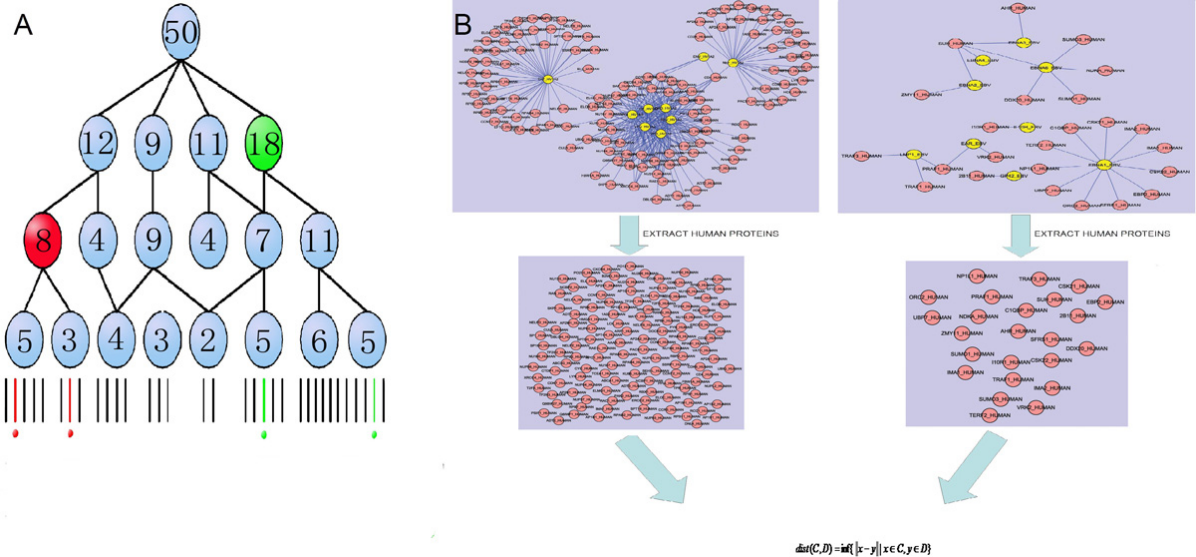
**HIV relationship tree based on molecular function**. All the virus IDs shown in the figure are Uniprot IDs. The length of each branch represents the distance between different viruses. HIV-1 and HIV-2 virus are almost completely separated, except HV1LA, which belongs to the HIV-1 group M subgroup B and is grouped with the HIV-2 virus, and HV2KR falls in the HIV-1 virus clade. SIVM1, which represents the simian immunodeficiency virus, shows closer relationship with HIV-2 virus. Meanwhile, HV192 has a little difference with other HIV-1 viruses in our figure. According to the graph, the distances among HIV-1 viruses are relatively farther than the distances between HIV-2 viruses.

Examination of HIV classification using our method reveals additional insights. HIV has two subtypes, HIV-1 and HIV-2, which are extracted from chimpanzees and sooty mangabeys respectively [20]. Both subtypes of HIV are transmitted by sexual contact, bodily fluid, or from mother to child. They can cause AIDS, and lead to the symptom of Immunodeficiency. The two subtypes cannot be distinguished without tests performed by a specialized physician. The viruses that belong to the Primate lentivirus group, which could be displayed as the representation of the HIV in our dataset, were selected out and integrated in Figure 2. As shown in the molecular function tree (Figure 2), almost all HIV-1 and HIV-2 viruses were divided into two categories (GO terms of human proteins that correspond to separate HIV-1 and HIV-2 are stored in Additional file 1). The only two exceptions were the HV1LA and HV2KR. The former represents an HIV-1 virus belonging to group M, and is grouped with the HIV-2, while HV2KR, which is defined as a Human immunodeficiency virus type 2 virus, is classified with the majority of HIV-1. Considering the subtle differences between the two subtypes of HIV, our result revealed the potential relationship and indicated the similarity between not only HV1LA and HIV-2 virus, but also HV2KR and HIV-1 virus. Meanwhile, SIVM1, which is regarded as simian immunodeficiency virus, was grouped closer with the HIV-2 viruses than HIV-1 viruses. In fact, previous study has proposed that a strain of SIV jumped from Sooty Mangabey to become the HIV-2 virus [21]. In addition, phylogenic methods also have shown the SIV in Sooty Mangabey and Macaque have close relationship to HIV-2 virus [22]. This evidence, to certain extent, strengthens the reliability of our method. Moreover, we had computed the relationship between different HIV viruses on the basis of 'Biological Process' and 'Cellular Component', and obtained very similar tree structures to the one based on 'Molecular Function' (Additional 2). The generally successful separation of HIV-1 and HIV-2 viruses in the molecular function tree demonstrated the feasibility of our method to evaluate the relationship between viruses.

In evolution research, different indicators, such as the similarity between conserved sequences, have been used to determine the distances between organisms. In our method, we have defined the smallest special score derived from the SSBP of proteins between two sets as the distances between different viruses. The mathematical definition of the distance between two sets is the infimum of the distance between any components of the two sets. Our definition of the distance between two viruses is consistent with the mathematical distance definition of two point sets. Moreover, some human proteins, with which a viral protein interacts, could exert their function in relatively general processes. These general proteins contribute less to differentiate viruses. In an ideal situation, we should use the proteins that have more specific functions and participate in more special processes to reflect the relationship between different viruses. The infimum represents the most specific similarity between two protein sets and could reflect the relationship between two viruses on the most specific level. Considering the definition of GO term is at a general level, the smallest special score also has the tendency to get rid of the non-specificity of some GO terms in our sets.

In our new approach, the ability to detect the relationship between distinct viruses relies on the quality of the virus-host protein-protein interaction network explicitly. If the network is reliable and contains enough information to bridge the connection between viruses and their hosts, the relationship disclosed based on PPI network would reveal more functional associations to the virologists who are interested in the relationships between different viruses. In total, 9683 human proteins are confirmed to interact with these viral proteins of 114 viruses, and among them 8249 human proteins are verified to interact with 48 HIV viruses, while 66 non-HIV viruses correspond only to 1434 human proteins. This number is relatively low compared to the number of human proteins that interact with HIV proteins. As discussed above, the classification of HIV viruses displayed much more reliable result than for the rest groups of the viruses. This might be caused by the difference between the amounts of data in the two corresponding datasets that are currently available. It is expected that more verified virus-host protein-protein interaction data of other viruses may lead to more reliable and valuable results for exploring potential relationships between distinct viruses. Our method points to a new direction to elucidate the relationship between viruses on the systematical level and provides rich information for virologists to study the relationships among various viruses.

## Materials and methods

### Protein-protein interaction network

The protein-protein interaction network used in this paper is constructed mainly according to the results of Dyer et al. [23], which focuses on the interaction between human protein and pathogen protein, and a database (http://molvis.vbi.vt.edu/pig/index.php) describing the pathogen and human protein-protein interaction. We combined the data from the two sources mentioned above to build the protein interaction networks between each virus and its host, human, in our paper. Each interaction pair in the network is composed of a viral protein and a human protein. This network is the foundation that we used to evaluate the relationships between different viruses. The distance between distinct viruses is calculated according to the functional similarity of the host protein that interacts with the corresponding viral protein in the network. In the GO system, each human protein has been normally assigned GO terms in three different categories - 'Cellular Component', 'Biological Process' and 'Molecular Function', we used the GO term - "Molecular Function" to carry out the analysis since researchers usually are more interested in the molecular function of a protein when they study the virus and host interaction.

### Distance between different viruses

**Figure 3:**
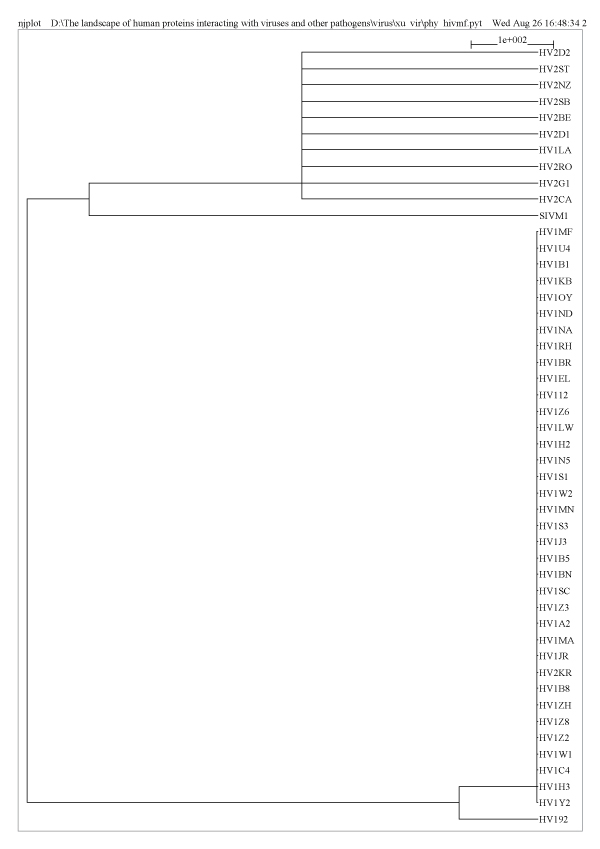
**The process of calculating the special score system derived from SSBP**. A. The definition of special score is similar to that of SSBP, where the red proteins share the parent term that the gene product concerning it is 8, while the counterpart of the green protein is 18. Thus, the red protein pair will have the score 8, meanwhile the green pair corresponds to18. The greater score of the green pair indicates less similarity than the red pair. B, The PPI network between not only human and HV1A2 but also human and EBV. The spot in the picture represents the protein, and the line that links two proteins stands for the interaction between them. The name of the protein is displayed on the spot, while the suffix of each name shows species to which this protein belongs. The human proteins are marked red while the viral proteins are marked yellow. As shown in the picture, the human protein in the protein-protein interaction network is selected out to form two sets, then the distance between the two sets are stored as the distance of the two viruses.

As some viruses exhibit co-evolution with their host [12,13], assessing the distance between different viruses according to the virus-host protein-protein interaction network is considered to be reasonable. The human proteins that interact with the proteins of a target virus are selected out from the network to form a set. As shown in Figure 3B, the subnetwork that contains the interaction pairs between human proteins and viral proteins from an identified virus (here corresponding to HV1A2 and EBV respectively) is extracted from the whole PPI network. The related interaction partners in human to each virus (red spot in Figure 3B) are selected to form the set. Each set is named after the related virus and represents the functional property of the virus in the network according to the "function association" theory. Next, the distance between different viruses is evaluated based on the functional similarity of the corresponding sets as follows: First, each component of a set is picked out to form a protein pair with each component of another set. Then, the special score, derived from the method of smallest shared biological process (SSBP), of each pair is calculated. The infimum (inf) of the score mentioned above is defined as the distance between the two corresponding viruses on the basis of the distance between the two sets in the field of mathematics (see formula 1). Then, the relation tree of different viruses is constructed based on the above resulted distance. In the end, the relationships among different viruses are inferred according to GO term based on the molecular function of host proteins participating in the virus and human interaction.

The distance between two sets C and D is defined as:

(1) $$dist\left(C,D\right)=\inf\left\{∥x-y∥|x\in C,y\in D\right\}$$

### The score system derived from SSBP

The method of smallest shared biological process, which is used to measure the functional similarity between different proteins in the previous work [24,25], is the reference of the special score system applied in this paper. The following procedure was used to quantify functional similarity between two proteins according to SSBP: First, identify all gene ontology terms shared by two proteins; next, count how many other proteins were assigned to each of the shared terms as well; finally, identify the shared biological process term with the smallest count (SSBP). The SSBP score between each protein-protein pair is recorded. As shown in the Figure 3A, the two numbers 8 and 18 in the red or green circles are the SSBP scores of the red protein pair and green protein pair, respectively. The special score system used in this article is generally derived from the SSBP. Instead of calculating the protein relationships based on their biological process GO terms, we focused on their relationships based on the GO terms in "Molecular Function" category. This is the only difference between our method and SSBP. Last, the infimum of the special score of two sets, which is described in the previous part, is stored in the distance matrix to measure the relationship between two viruses.

## Competing interests

The authors declare that they have no competing interests.

## Authors' contributions

Data collection: FX. Programming: FX, CZ, LJ. Design of the analysis process: TLS, YD and FX. Data analysis: FX, LJ, TLS. Paper Writing: FX, YHL, TLS.

## Supplementary Material

Additional file 1 **This file contains the GO terms of human proteins that correspond to separation of HIV-1 and HIV-2**. Click here for file

Additional file 2 **Tree files based on the GO terms of "Biological Process", "Cellular Component" of HIV and all of the viruses in our network have been stored in this supplementary file**. These tree files can be viewed with the help of "treeview" and other similar software. Click here for file
